# Short-term effects of pesticide fipronil on behavioral and physiological endpoints of *Daphnia magna*

**DOI:** 10.1007/s11356-021-13091-6

**Published:** 2021-02-26

**Authors:** Adam Bownik, Aleksandra Szabelak

**Affiliations:** Department of Hydrobiology and Protection of Ecosystems, University of Life Sciences in Lublin, Dobrzańskiego 37, 20-262 Lublin, Poland

**Keywords:** Behavior, Crustacean, Insecticides, Motility, Physiological activity

## Abstract

Fipronil (FIP) is an organic pesticide with many practical uses. Although some results indicated toxic effects in some terrestrial and aquatic animal species, little is known on its influence on behavioral and physiological endpoints of cladocerans. The aim of our study was to determine the short-term effects of FIP at concentrations of 0.1 μg/L, 1 μg/L, 10 μg/L, and 100 μg/L on *Daphnia magna* sublethal indices: behavioral (swimming speed, distance traveled) and physiological endpoints (heart rate, post-abdominal claw activity and thoracic limb movements). The results showed that FIP induced reduction of swimming speed and distance traveled in a concentration- and time-dependent manner at all the concentrations used. The lowest concentration of the insecticide temporarily stimulated post-abdominal claw activity after 24 h and thoracic limb activity after 48 h; however, the highest concentrations reduced all the studied physiological endpoints. IC50 values showed that thoracic limb activity, swimming speed, and distance traveled were most sensitive to FIP after 24-h exposure. The most sensitive parameter after 48 h and 72 h was swimming speed and post-abdominal claw activity, respectively. The study indicated that (i) behavioral and physiological endpoints of *Daphnia magna* are reliable and valuable sublethal indicators of toxic alterations induced by FIP; however, they respond with different sensitivity at various times of exposure, (ii) FIP may alter cladoceran behavior and physiological processes at concentrations detected in the aquatic environment; therefore, it should be considered as an ecotoxicological hazard to freshwater cladocerans.

## Introduction

Fipronil (FIP), a derivative of phenylpyrazole, is one of the most widely used organic pesticides in agriculture, veterinary medicine, and urban households for selective elimination of insect pests such as ants, mosquitos, termites, cockroaches, spiders, fleas, and mites (Tomlin 1994; Aajoud et al. 2003; Scarampella et al. 2005; Gunasekara et al. 2007; MacLachlan 2008; Simon-Delso 2014; Ardeshir et al. 2017; Rust 2017). The mechanism of FIP toxicity is, unlike most of commonly used insecticides, blocking the receptors responsible for binding γ-aminobutyric acid (GABA) and glutamic acid of the chlorine channels ions located in the nerve cells and, as a consequence, inducing the overexcitation of the nervous system leading to paralysis, and finally death (Hossie et al. 1995; Bloomquist 2003; Gunasekara et al. 2007). Blocking of chlorine channels is much more effective in insects than in vertebrates (Bonmatin et al. 2015); therefore, FIP is assumed to be more toxic to insects than mammals.

Massive use of FIP for veterinary purposes led to hazardous incidents. Eradication of red mites in poultry farms in European countries and Japan (Van Poucke et al. 2016; Hatta et al. 2019) induced transfer of FIP to hen tissues and eggs (European Food Safety Authority (EFSA) et al. 2018; Charalampous et al. 2019). The presence of FIP in food is especially important for human health as this pesticide was classified by the US EPA as a group C (possible human) carcinogen (Charalampous et al. 2019). Because of wide application of FIP in agriculture, it is detected in terrestrial, marine, and water environments (Ngim and Crosby 2001; Bonmatin et al. 2015). FIP is a stable compound with half-life exceeding 100 days at a temperature of 25°C, and it may be easily transferred to surface water (Bonmatin et al. 2015). Fipronil is stable to hydrolysis at pH 5.5 and pH 7, but at pH 9, its hydrolytic half-life is 28 days (Macbean 2012). This pesticide has been detected in various aquatic ecosystems such as estuaries, urban waterways, and lakes (Ensminger et al. 2013; Weston and Lydy 2014; Wu et al. 2015) in the range of 0.5–10.004 μg/L (Gan et al. 2012; Ruby 2013; Wu et al. 2014; Michel et al. 2016). The presence of FIP and its sulfone and sulfide metabolites was detected in urban streams in several states of the USA (Demcheck and Skrobialowski 2003). For example, in the USA, FIP was found at a concentration of 0.117 μg/L in Louisiana, while the levels of its metabolites FIP-sulfone, FIP-sulfide, FIP-desulfinyl, and FIP-amide were 0.038 μg/L (Colorado), 0.015 μg/L, (Louisiana), 0.158 μg/L (California), and 0.011 μg/L (Louisiana), respectively (US Geological Survey 2006). In the runoff water of California, FIP was found at the range of 204–1170 ng/L (Gan et al. 2012). The above-mentioned levels found in aquatic environments exceed the acute and chronic aquatic life benchmark concentrations of FIP (0.1 μg/L and 0.01 μg/L, respectively) in water suggested by the US EPA (Stone et al. 2014).

A number of studies showed various detrimental effects induced by FIP in terrestrial non-target insects such as honeybees and midges (Apenet 2011; Holder et al. 2018; Monteiro et al. 2019). It was indicated that this pesticide inhibits feeding activity of dragonflies (Jinguji et al. 2018). Other terrestrial animals such as earthworms, amphibians, rabbits, and certain groups of gallinaceous birds were also reported to be affected by FIP (Pisa et al. 2015).

Since FIP is transferred to water reservoirs, it is available to aquatic biota. Degradation products of FIP may have higher toxicity, and they may be more stable than FIP itself Gunasekara et al. 2007; Tingle et al. 2003). Environmental conditions such as sunlight, acidity, temperature, microbial community composition, and presence of additional substances may modulate the overall toxicity of FIP in aquatic ecosystems (Bonmatin et al. 2015). It was noted that in increased salinity, FIP and its photodegradation products such as desulfinyl metabolite may be more bioavailable to benthic organisms (Goff et al. 2017); therefore, marine organisms should be considered as particularly susceptible to intoxication. Various effects induced by FIP were found in aquatic vertebrates and invertebrates (Schlenk et al. 2001; Wirth et al. 2004; Pisa et al. 2015; Gibbons et al. 2015; Charalampous et al. 2019). Some authors indicated that this compound is highly toxic to zebrafish larvae (Xu et al. 2018; Park et al. 2020) and a fish species *Aristichthys nobilis*, possibly due to high affinity of GABA receptors (Zhang et al. 2018). In zebrafish embryos, larvae and adults adverse changes of behavioral parameters and oxidative stress were also found (Stehr et al. 2006; Bevilaqua et al. 2020). FIP also induces toxic effects in marine and freshwater crustaceans such as estuarine copepod *Amphiascus tenuiremis* (Chandler et al. 2004), kuruma prawn, sand shrimp, and a surrogate mysid species *Americamysis bahia* (Hano et al. 2019), blue crab *Callinectes sapidus* (Goff et al. 2017), juvenile brown shrimp *Farfantepenaeus aztecus* (Al-Badran et al. 2018), and grass shrimp *Palaemonetes pugio* (Key et al. 2003).

*Daphnia* sp. is a keystone freshwater crustacean playing many important ecological roles. They are filter feeders regulating the number of various microorganisms such as algae, cyanobacteria, protozoans, and small particles suspended in water reservoirs (Stollewerk 2010). Moreover, they are a food source for some predator species. Daphnids are also a common model organism used in acute and chronic toxicity tests (Zhang et al. 2016). Most of the available studies on the effects of FIP on cladocerans deal with determination of lethality and immobilization of model daphnids (Konwick et al. 2005; Stark and Vargas 2005; Hayasaka et al. 2012). However, detection of sublethal alterations induced by FIP may require more sensitive endpoints. Currently, novel approaches using reliable and early indicators such as behavioral (e.g., swimming speed) and physiological endpoints (e.g., heart rate, thoracic limb activity) have been introduced in ecotoxicological testing. Scientific literature indicates that alterations of these parameters may be detected much earlier before the experimental organisms are immobilized or killed (Huang et al. 2017, 2018; Bownik 2017; Bownik et al. 2020a). Therefore, the aim of our study was to investigate whether FIP affects behavioral (swimming speed and distance traveled) and physiological endpoints (the heart rate, post-abdominal claw activity, and thoracic limb activity) of *Daphnia magna*.

## Material and methods

### Animal culture and FIP preparation

*Daphnia magna* were obtained from a single mother hatched from a dormant egg according to the procedure by Microbiotest (Belgium) (Persoone et al. 2009). The animals were cultured in 5 L of medium at a temperature of 23 ± 2°C, under light/dark period of 16:8 h. The medium was prepared in accordance with ASTM standards (American Society of Testing and Materials 1986). The animals were fed once daily with a few drops of powdered *Spirulina* (2 mg/L water) per tank and supplemented with a few drops of baker’s yeast (10 mg/L per tank).

FIP (analytical standard, of > 97% purity) was purchased from Merck, and the following series of its nominal concentrations was prepared: 0.1 μg/L, 1 μg/L, 10 μg/L, and 100 μg/L. Initially, the stock solution (100 mg/L) was prepared by diluting 10 mg of FIP in the 100 mL of filtered culture medium containing 0.1% of methanol. After mixing the flask with the stock solution, the highest FIP working concentration of 100 μg/L was prepared by transferring 100 μL of the stock solution to a volumetric flask containing 99.9 mL of clean culture medium. The experimental concentration of 10 μg/L was done by transferring 10 mL from the flask with 100 μg/L of FIP to another volumetric flask containing 90 mL of clean medium. A concentration of 1 μg/L was prepared by transferring 10 mL from the flask containing 10 μg/mL of FIP to another flask with 90 mL of clean medium. A concentration of 0.1 μg/L was prepared by transferring 10 mL from the flask containing FIP at 1 μg/mL to another flask with 90 mL of clean medium. The appropriate solutions of the insecticide prepared in the above-mentioned flasks were transferred at a volume of 8 mL to glass Petri dishes (75 mm of diameter). Each studied endpoint was examined at 24 h, 48 h, and 72 h of the exposure. Animals were considered as dead when no heart activity was noted under microscopic examination.

### Swimming speed and distance traveled

Each experimental group consisted of 15 neonates grouped into 5 individuals placed in three glass Petri dishes (for three independent treatments) with a diameter of 75 mm containing one of FIP concentrations of 0.1 μg/L, 1 μg/L, 10 μg/L, and 100 μg/L. Additionally, 15 animals grouped into 5 individuals placed in three experimental dishes with medium only were treated as the control. Daphnids were left 15 min for acclimation (Bownik and Pawlik-Skowrońska 2019). Horizontal swimming was recorded for 1 min in each Petri dish (starting from the animals exposed to the highest concentration of FIP) with a digital camera Nikon D3100 mounted on a stable stand (Bownik et al. 2019a, b, 2020a) at 24 h, 48 h, and 72 h of exposure. The recorded video files were then analyzed by frame-by-frame method with Tracker® 5.1.3 computer software. The swimming tracks of a single animal were recorded by clicking the cursor on the daphnid image in the separate frames of the video clip. The swimming tracks (interpreted by the program as a mass point) were plotted on the screen, and the mean speed (v) was calculated by the software and expressed in millimeters per second (mm/s). Distances traveled by the individuals were also calculated by Tracker® 5.1.0 software after matching the swimming trails.

### Determination of physiological endpoints

Heart rate, post-abdominal claw activity, and thoracic limb movement were determined by a microscopic method supported by previously described digital video analysis (Campbell et al. 2004; Bownik et al. 2019a, b, 2020b). Briefly, individual daphnids were gently transferred with a Pasteur pipette in about 50-μL drop from each experimental Petri dish or the control group to a microscope slide for the microscopic analysis. The microscopic view of the examined daphnid was recorded for at least 1 min (with the speed of 30 frames per second) with a digital camera Nikon D3100 mounted on a light microscope. The magnification (×30–100) and camera resolution allowed performing the analysis with a good visibility of the heart. The physiological endpoints were calculated with a frame-by-frame method supported by a video player by counting the number of beats (heart and thoracic limbs) or movements (post-abdominal claw) per 1 min.

### Determination of IC50

Effective (nominal) concentration (EC50) for immobilization and inhibition (nominal) concentration (IC50) for swimming speed, distance traveled, heart rate, thoracic limb activity, and post-abdominal claw activity after 24 h, 48 h, and 72 h of exposure were calculated using probit regression analysis with MedCalc 19.3.1 statistical software (MedCalc—version 19.3.1. MedCalc Ltd.). Maximum log likelihood estimation (null model -2 Log likelihood, full model -2 Log likelihood) was used as an overall fit model, and logarithmic curve was plotted as a result.

### Statistical analysis

Data were analyzed with statistical software Statistica® 13.1. Normality and homogeneity of variances were estimated by the Shapiro-Wilk and Levene’s tests, respectively. The tests were parametric with homogenous data. The comparisons of means among the experimental groups were done by two-way ANOVA (interactions: concentrations, time and time*concentration) followed by Dunnett’s post hoc test to calculate significant differences between the experimental groups and the control. Results were significant when *p* < 0.05. The results are presented as means ± standard deviation (SD).

## Results

### EC50 and IC50 for *Daphnia magna* endpoints

**Fig. 1:**
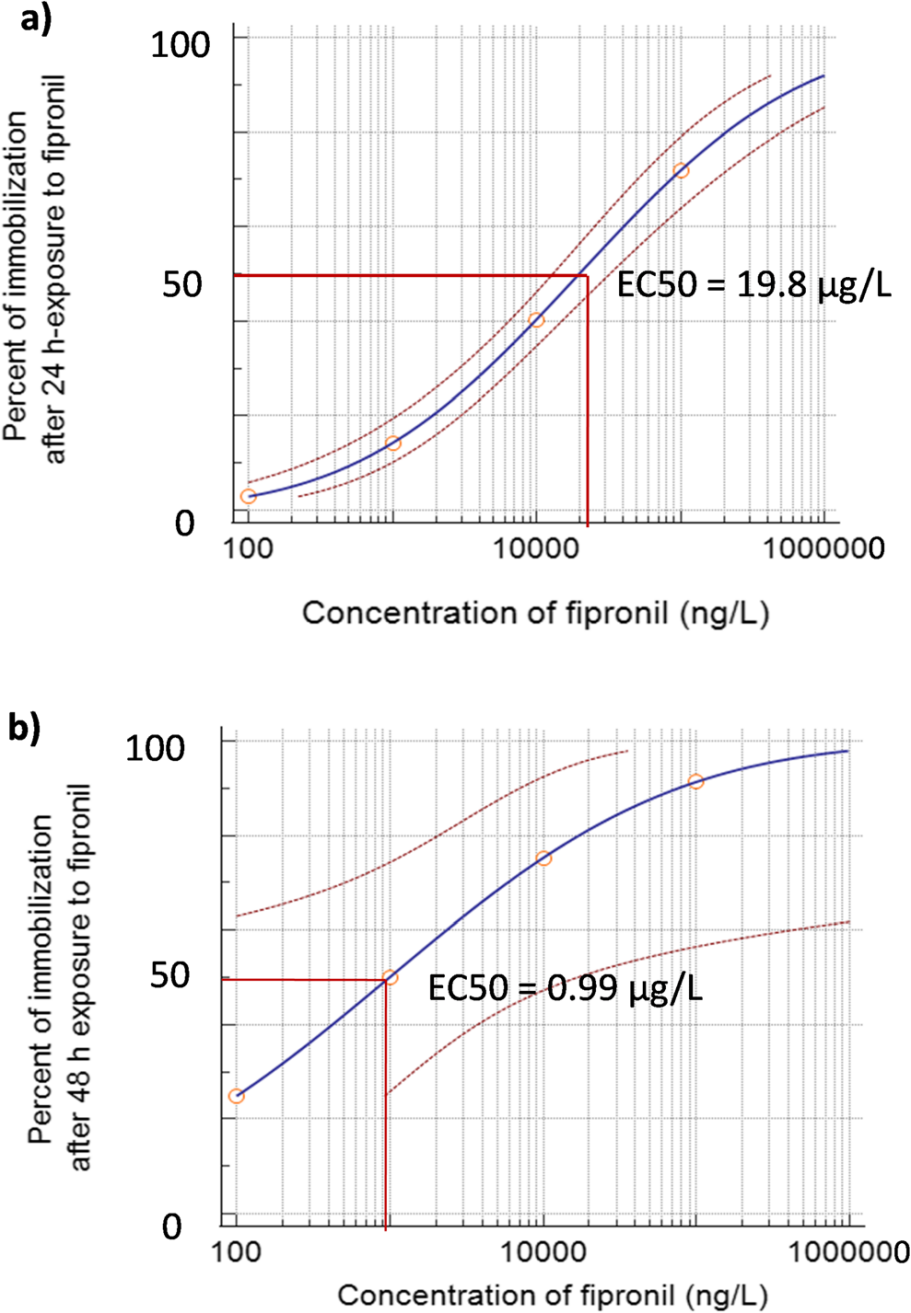
Logarithmic curves of effective (nominal) concentration (EC50) 24-h (**a**) and 48-h exposure (**b**) for *Daphnia magna* immobilization after exposure to fipronil obtained by probit regression analysis. Gray vertical dashed lines represent logarithm scale of fipronil nominal concentration

Calculation of nominal IC50 of the studied endpoints revealed that the most sensitive endpoints after 24-h exposure to FIP were thoracic limb activity (IC50 = 2.8 ± 0.7 μg/L) (Table 1; Fig. 1), swimming speed (3.5 ± 0.2 μg/L), and distance traveled (3.3 ± 0.2 μg/L). Heart rate was the least sensitive indicator (708 ± 27 μg/L); however, its EC50 value after 72-h exposure was similar to those of the behavioral endpoints. EC50 for daphnid immobilization showed the lowest sensitivity after 72 h of exposure (0.5 ± 0.04 μg/L).

**Table 1:** Nominal EC50 values (μg/L) for immobilization and IC50 values (μg/L) for the studied behavioral and physiological endpoints of *Daphnia magna* after 24-h, 48-h, and 72-h exposure to fipronil

Time	Immobilization (EC50)	IC50 (μg/L)				
		Swimming speed	Distance traveled	Heart rate	Post-abdominal claw activity	Thoracic limb activity
24 h	*19.8 ± 0.3* (12.9–32.2)	*3.5 ± 0.2* (1.1–11.6)	*3.3 ± 0.2* (1.0–11.0)	*708 ± 27* (162.0–1288.0)	*17.8 ± 0.3* (1.4–34.3)	*2.8 ± 0.7* (1.2–6.2)
48 h	*1.0 ± 0.06* (0.02–16.6)	*0.4 ± 0.07* (0.1–1.4)	*0.5 ± 0.06* (0.1–1.5)	*4.5 ± 0.3* (2.1–9.5)	*0.6 ± 0.07* (0.03–5.8)	*1.6 ± 0.1* (0.8–3.3)
72 h	*0.5 ± 0.04* (0.2–0.7)	*0.2 ± 0.1* (0.1–0.5)	*0.2 ± 0.1* (0.1–0.5)	*0.2 ± 0.1* (0.1–0.6)	*0.04 ± 0.01* (0.01–0.1)	*0.09 ± 0.01* (0.4–1.4)

Lower and upper 95% confidence intervals are given in parentheses, *p* < 0.0001

### Immobilization

**Fig. 2:**
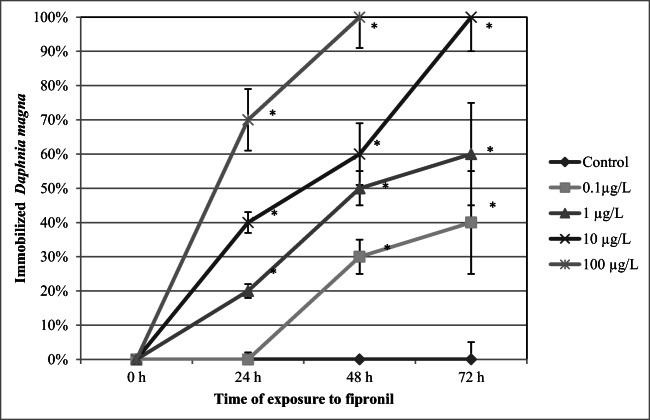
Immobilization (%) of *Daphnia magna* exposed to various concentrations of fipronil. Results are presented as means ± SD, *n* = 15; * - statistical significance when compared to the control at *p < 0.05; **p < 0.01

The results showed that *Daphnia magna* exposed to FIP showed concentration- and time-dependent immobilization (Fig. 2; Table 1). After 48 h, immobilization was observed at all the concentrations of the compound use in the study (30 ± 5%, 50 ± 5%, 60 ± 9%, and 100 ± 9% at 0.1 μg/L, 1 μg/L, 10 μg/L, and 100 μg/L, respectively). The highest percentage of the immobilized animals was noted after 72 h of the exposure (40 ± 9%, 60 ± 15%, and 100 ± 10% at 0.1 μg/L, 1 μg/L, 10 μg/L, respectively).

### Swimming speed

**Fig. 3:**
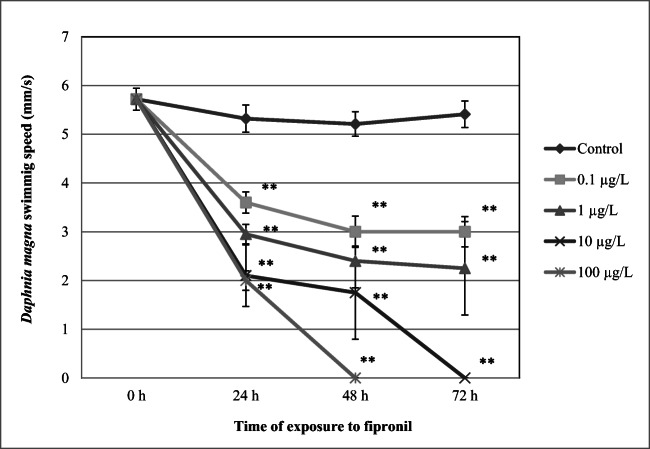
Swimming speed of *Daphnia magna* exposed to various concentrations of fipronil. Results are presented as means ± SD, *n* = 15; * - statistical significance when compared to the control at *p < 0.05; **p < 0.01

Effects of FIP on daphnids swimming speed are presented in Fig. 3. The result show that the insecticide induced a substantial decrease of daphnid swimming speed at all tested concentrations after 24 h (3.60 ± 0.21 mm/s, *p* < 0.01; 2.95 ± 0.20 mm/s, *p* < 0.01; 2.1 ± 0.63 mm/s, *p* < 0.01; 2.0 ± 0.20 mm/s, *p* < 0.01 at 0.1 μg/L, 1 μg/L, 10 μg/L, 100 μg/L, respectively) when compared to the control (5.32 ± 0.22 mm/s). More reduced parameter was found in the treated animals after 48 h, and complete cessation of motility was noted in the crustaceans exposed to 100 μg/L after 48 h and 10 μg/L after 72 h.

### Distance traveled

**Fig. 4:**
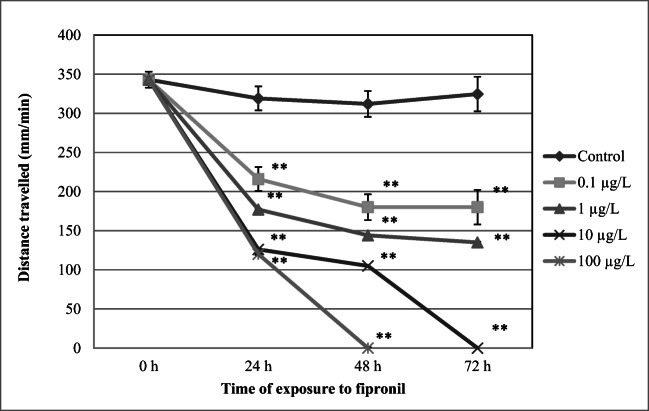
Distance traveled by *Daphnia magna* during exposure to fipronil. Results are presented as means ± SD, *n* = 15; * - statistical significance when compared to the control at *p < 0.05; **p < 0.01

Daphnids exposed to FIP showed a concentration- and time-dependent reduction of distance traveled (Fig. 4). A distinct inhibition of this endpoint was noted after 24 h in the group of crustaceans treated with the lowest concentration of 0.1 μg/L (216 ± 37 mm/min; *p* < 0.01) when compared to the control (319 ± 15 mm/min). The distance traveled was decreasing with the increasing time of exposure to FIP. No distance traveled was noted in daphnids at 100 μg/L after 48 h and at 10 μg/L after 72 h.

### Heart rate

**Fig. 5:**
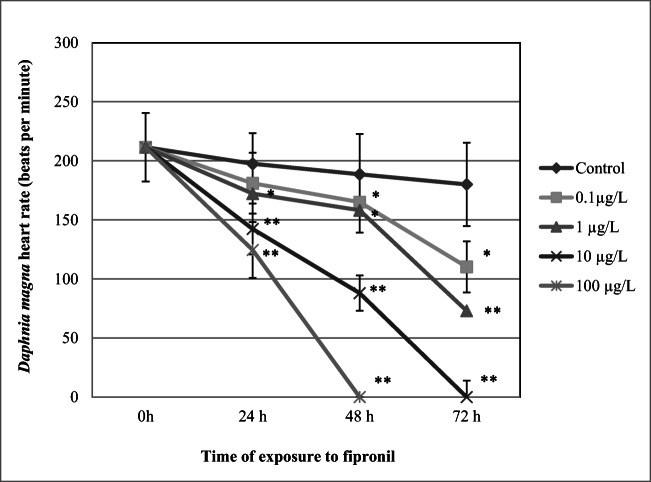
Heart rate of *Daphnia magna* exposed to fipronil. Results are presented as means ± SD, *n* = 15; * - statistical significance when compared to the control at *p < 0.05; **p < 0.01

The results show that FIP at concentrations of 1 μg/L, 10 μg/L, and 100 μg/L reduced daphnid heart rate after 24-h exposure (172.2 ± 25.7 beats per minute [bpm], 142 ± 21.5 bpm, *p* < 0.01, and 124 ± 26.6 bpm, *p* < 0.01, respectively), when compared to the control (197.5 ± 25.8 bpm) (Fig. 5). Further exposure resulted in more spectacular reduction of daphnid heart rate. After 72-h treatment with FIP, the endpoint was 110 ± 21.8 bpm at 0.1 μg/L, 73 ± 13.9 bpm *p* < 0.01 at 1 μg/L, and 0 ± 0 bpm *p* < 0.01 at 10 μg/L when compared to the control (180 ± 35 bpm).

### Post-abdominal claw activity

**Fig. 6:**
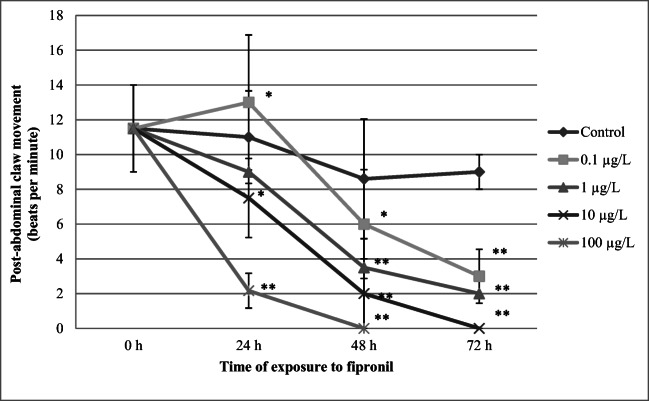
Post-abdominal claw activity of *Daphnia magna* exposed to various concentrations of fipronil. Results are presented as means ± SD, *n* = 15; * - statistical significance when compared to the control at *p < 0.05; **p < 0.01

The results presented in Fig. 6 show that FIP at a concentration of 0.1 μg/L induced a transient increase of post-abdominal claw activity. However, a decrease of this endpoint was found at concentrations of 10 μg/L (7.5 ± 2.2 bpm; *p* < 0.01) and 100 μg/L (2.2 ± 0.9 bpm; *p* < 0.01) after 24-h exposure when compared to the control (11.5 ± 2.5 bpm). The highest reduction of this physiological activity showed daphnids at 72-h exposure to the pesticide (3 ± 1.5 bpm, *p* < 0.01; 2 ± 0.2 bpm, *p* < 0.01; and 0 ± 0 bpm *p* < 0.01) at concentrations of 0.1 μg /L, 1 μg/L, 10 μg/L, respectively).

### Thoracic limb activity

**Fig. 7:**
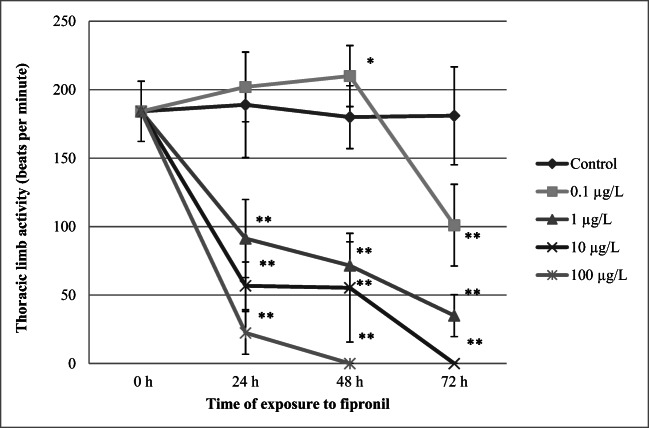
Thoracic limb activity of *Daphnia magna* exposed to various concentrations of fipronil. Results are presented as means ± SD, *n* = 15; * - statistical significance when compared to the control at *p < 0.05; **p < 0.01

Figure 7 shows that the activity of daphnid thoracic limbs was increased significantly after 48 h of the exposure to FIP at a concentration of 0.1 μg/L (210 ± 23 bpm) when compared to the control (180 ± 22 bpm). However, higher concentrations of the pesticide induced a concentration- and time-dependent decrease of this endpoint. More significant reduction was found in daphnids exposed to a concentration as low as 1 μg/L (72 ± 17 bpm, *p* < 0.01) both after 48 h (35 ± 15 bpm, *p* < 0.01) and after 72 h of exposure.

## Discussion

Little is known on sublethal effects of FIP in crustaceans. Lethal effects of FIP in crustaceans were documented by some authors. For example, it was revealed that the LC50 value after 48-h exposure of *Daphnia pulex* was estimated to be 0.0156 mg/L (Stark and Vargas 2005). FIP was also found to be lethal to brown shrimp (*Farfantepenaeus aztecus)* (96-h LC50 = 0.12 μg/L) (Al-Badran et al. 2019) and a marine grass shrimp *Palaemonetes pugio* (96-h LC50 = 0.32 μg/L) (Overmyer et al. 2007). Some sublethal effects of FIP on *Ceriodaphnia silvestrii* life history traits, growth of brown shrimp *Farfantepenaeus aztecus*, and reproduction of copepods were also found (Cary et al. 2004; Al-Badran et al. 2019; Moreira et al. 2020). The present study showed that FIP induced toxic changes of *Daphnia magna* endpoints in a concentration- and time-dependent manner. Exposure of experimental animals to FIP resulted in immobilization observed after 24-h and 48-h exposure with nominal EC50 values of 19.8 ± 0.3 μg/L and 1.0 ± 0.06 μg/L, respectively. Inhibition of daphnid motility was also observed by other authors; however, Hayasaka et al. (2012) found that higher concentration of FIP (88.3 μg/L) was required to induce 50% immobilization of *Daphnia magna* after 48 h of exposure. Since pure analytical standard was used in the present experiments and a commercial formulation with an active ingredient in the study performed by Hayasaka et al. (2012), different EC50 values may result from different purities of FIP used in both experiments. Immobilization induced by FIP was also found in other cladocerans. However different EC50 values were obtained by various authors. Although 48-h EC50 of FIP were 0.99 μg/L and 8.83 μg/L for immobilization of *Ceriodaphnia dubia* and *Ceriodaphnia reticulata*, respectively (Hayasaka et al. 2012), Konwick et al. (2005) calculated the 48-h EC50 for *Ceriodaphnia dubia* to be as high as 10.3 μg/L. Some authors documented sublethal effects were found in crustaceans such as male infertility decreased reproductive rate, birth rate an increase in generation time (Cary et al. 2004; Stark and Vargas 2005; Moreira et al. 2020). Our calculation of nominal IC50 for *Daphnia magna* showed that both behavioral and physiological endpoints are more sensitive to FIP than immobilization because the IC50 of behavioral parameters were much lower than EC50 for immobilization after 24 h. Thoracic limb activity, swimming speed, and distance traveled turned out to be the most sensitive endpoints to FIP. Swimming speed was the most sensitive after 48 h. However, after 72 h of the exposure thoracic limb activity again showed the lowest IC50 value. Although heart rate was the least sensitive after 24 h, the IC50 was comparable with the other endpoints after 72 h.

This study showed that FIP induced a concentration- and time-dependent decrease of daphnid swimming speed which may be explained by neurotoxic activity of the pesticide. It is possible that blocking the GABA-regulated chloride channels in neurons resulted in overexcitation of neurons innervating daphnid muscles of second antennae responsible for swimming activity which led to spastic paralysis as it was found in insects (Hossie et al. 1995). Similar results were obtained by Al-Badran et al. (2018) who found reduction of locomotor activity in juvenile brown shrimp *Farfantepenaeus aztecus* with specific movement disturbances such as swimming in circles. Our results also seem to be in agreement with those obtained by Hussain et al. (2020) who demonstrated depression of *Daphnia magna* locomotor activity after the exposure to FIP at a concentration of 0.117 μg/L. Alterations of motility was also noted in fish exposed to FIP; however, changes were associated with increased swimming activity (Gibbons et al. 2015; Wang et al. 2016; Hussain et al. 2020). The opposite effects on swimming behavior in *Daphnia magna* and fish may be attributed to differences in the mechanisms related with the release of neurotransmitters in synapses after direct interaction of FIP with receptors for GABA or glutamic acid (Zheng et al. 2014).

Distance traveled by daphnids is a behavioral parameter which indicated toxic effects induced by various agents (Jang et al. 2016; Bownik 2017). Our study showed that the distance traveled by the experimental animals was decreasing with increasing concentration of FIP which was manifested by the shorter tracks of daphnids exposed to the highest compound concentration when compared to the control. Similar results were obtained by Hussain et al. (2020) who noted reduction of distance traveled by *Daphnia magna* and zebrafish larvae exposed to FIP. Reduction of swimming distances clearly indicates that *Daphnia* neuromuscular system is sensitive to FIP. Decrease of this parameter was also observed in fish (*Danio rerio*) exposed to FIP (Wu et al. 2015). Behavioral disorders may have ecological consequences for cladocerans such as disturbances of vertical and horizontal migration and higher susceptibility to predators.

Daphnids have a transparent carapace allowing clear observation of heart physiological activity (Campbell et al. 2004). We found that FIP induced a concentration- and time-dependent reduction of the number of heart contractions in *Daphnia magna*. It is possible that decrease of heart rate may result from a direct interaction of FIP with receptors for GABA and glutamic acid in neurons regulating daphnid cardiac activity. The knowledge on the effects of FIP on the activity of crustacean heart is scarce; however, some authors reported that this pesticide affects heart activity of bees (Nicodemo et al. 2014). The authors linked the disturbances of heart functioning with the inhibited mitochondrial activity resulting from ATP depletion. Toxicity of heart mitochondria by FIP were also found in rats (Seydi et al. 2019). On the basis of the above-mentioned studies, it may be hypothesized that disturbances of daphnid heart rate may be a result of mitochondrial dysfunctions. FIP was also noted to induce cardiotoxic changes in zebrafish larvae such as heart beat irregularity and disruption of blood vessel formation (Park et al. 2020). Dysfunctions of heart activity in *Daphnia magna* may have serious physiological consequences such as depressed hemolymph circulation.

Daphnid post-abdominal claw is an organ which removes the excess of particles absorbed by thoracic limbs (Young et al. 1997). Some results indicated that its activity may be altered by toxic chemicals at sublethal levels (Lovern et al. 2007; Bownik and Pawlik-Skowrońska 2019). Our study showed that FIP at the lowest concentration increased the post-abdominal claw activity after 24 h. This hormetic effects may be explained by the fact that lower concentrations of FIP may trigger slight neurostimulation related with increased activity of excitatory neurotransmitters. On the other hand, higher concentrations of FIP may evoke overexcitation of neuron innervating muscles of the post-abdominal claw leading to muscle paralysis (Pisa et al. 2015). Exposure of daphnids to FIP in natural conditions may lead to disturbances of feeding.

Thoracic limbs play an important role in daphnid feeding and ventilation (Pirow et al. 1999). Rhythmic beating of these appendages forms water currents carrying food and providing oxygen to the organism (Lari et al. 2017). Thoracic limb activity is a valuable toxicological endpoint useful in recognition of early signs of behavioral and physiological disorders induced by various detrimental factors (Lari et al. 2017; Bownik et al. 2019a, b). Little is known about the effects of FIP on thoracic limb activity of cladocerans; however, some authors revealed other insecticides such as lambda-cyhalothrin (Bownik et al. 2019a) or cypermethrin (Friberg-Jensen et al. 2010) may alter functioning of this organ. The present study showed that the lowest concentration of FIP increased the activity of thoracic limb movement. On the other hand, higher levels of the pesticide decreased the number of beats. The mechanism of depressive effects of FIP on thoracic limb activity may be related with overexcitation of neurons innervating daphnid thoracic limbs leading to excessive appendage muscle contraction. Since thoracic limbs are vital for daphnid feeding and ventilation, disturbances in functioning of these organs may affect food and oxygen supply. Natural exposure to FIP may be one of factors inducing disturbances in water filtration implicating the increase of the remaining biomass (bacteria, algae, protozoans) in aquatic reservoirs. The consequence of this phenomenon may be increased water turbidity. Ventilation deficits resulting from daphnid exposure to FIP may inhibit oxygen supply leading to hypoxia, metabolic disorders, and physiological and behavioral disturbances. Although little is known on the effects of FIP on cladoceran feeding activity in cladocerans, some authors found reduction of feeding ability in dragonflies (*Sympetrum* spp.) (Jinguji et al. 2018).

## Concluding remarks

The present study showed that daphnid behavioral and physiological endpoints are sensitive indicators of toxic changes induced by FIP; however, sensitivity of these responses was different at various times of exposure. Since FIP induced alterations of daphnid endpoints at environmentally relevant concentrations, it may be expected that vital behavioral and physiological processes may be impaired by this pesticide in the real scenario. More studies in daphnids are required that would provide results useful in explanation of the neurological mechanisms responsible for the behavioral and physiological alterations.

## Data Availability

The datasets used and/or analyzed during the current study are available from the second author on the reasonable request.
